# Sella Turcica Shape as a Marker for Breed and Sex Classification in Sheep

**DOI:** 10.3390/vetsci13030290

**Published:** 2026-03-19

**Authors:** Eylem Bektaş Bilgiç, Tomasz Szara, Ozan Gündemir, Zuzanna Kaska, Muhammed Taha Temir, Barış Can Güzel, Fatma İşbilir, Emine İrem Deveci, Alexandra-Andreea Cherșunaru, Mihaela-Claudia Spataru

**Affiliations:** 1 Department of Surgery, Faculty of Veterinary Medicine, Istanbul University-Cerrahpasa, Istanbul 34320, Türkiye; eylem.bilgic@iuc.edu.tr; 2 Department of Morphological Sciences, Institute of Veterinary Medicine, Warsaw University of Life Sciences-SGGW, 02-776 Warsaw, Poland; 3 Department of Anatomy, Faculty of Veterinary Medicine, Istanbul University-Cerrahpasa, Istanbul 34320, Türkiye; 4 Veterinary Clinic A-Z WET, Zamiejska 28, 03-580 Warszaw, Poland; zuzannakaska1@gmail.com; 5 Department of Veterinary Radiology, Faculty of Veterinary Medicine, Istanbul University-Cerrahpasa, Istanbul 34320, Türkiye; tahatemir@iuc.edu.tr; 6 Department of Anatomy, Faculty of Veterinary Medicine, Siirt University, Siirt 56100, Türkiye; baris.guzel@siirt.edu.tr (B.C.G.); fatmaisbilir42@gmail.com (F.İ.); 7 Department of Wild Animal Disease and Ecology, Faculty of Veterinary Medicine, Istanbul University-Cerrahpasa, Istanbul 34320, Türkiye; emine.deveci@iuc.edu.tr; 8 Faculty of Veterinary Medicine, Ion Ionescu de la Brad Iasi University of Life Science, 700489 Iasi, Romania; alexandra.chersunaru@iuls.ro (A.-A.C.); mihaela.spataru@iuls.ro (M.-C.S.)

**Keywords:** centroid size, computed tomography, geometric morphometrics, osteological collection, pituitary fossa, sheep breeds, skull base, three-dimensional modeling

## Abstract

Recent anatomical and morphometric studies indicate that the sella turcica is a structurally informative region and a distinctive anatomical formation that can exhibit shape variation among individuals. The aim of this study was to evaluate, in three dimensions, the extent to which sella turcica morphology differs among three sheep breeds (Akkaraman, Morkaraman, Zom) and between sexes. A total of 102 specimens were examined. All skulls were CT-scanned specifically for this study; the sella turcica region was reconstructed as a three-dimensional model, and 12 anatomical landmarks were manually digitized for each specimen. The findings showed that sella turcica size differed among breeds, with the Zom group exhibiting the largest sella turcica size. In contrast, no clear size difference was observed between females and males. Shape assessment also revealed differences among breeds, largely driven by the separation of Zom from Akkaraman and Morkaraman, whereas no distinct sex-related shape pattern was detected. Importantly, the breed-related shape differences persisted after accounting for size effects. Overall, these results suggest that the sella turcica carries a breed-associated morphological signal in sheep, while showing no pronounced sexual differentiation in the present sample.

## Simple Summary

The sella turcica is a small bony region at the base of the skull that forms the pituitary gland’s seating, which helps control growth, reproduction, and metabolism. In this study, we examined whether the shape and size of the sella turcica differ among three sheep breeds (Akkaraman, Morkaraman, and Zom) and between females and males. We scanned 102 skulls using computed tomography, which allowed us to create three-dimensional models of the sella turcica without breaking the bones, and then compared these models across groups. We found that the sella turcica differed among breeds, with Zom showing the clearest differences from the other two breeds. In contrast, females and males did not show clear differences.

## 1. Introduction

Akkaraman and Morkaraman are two major indigenous, fat-tailed sheep breeds of Türkiye that have been shaped by long-term, low-input management under continental steppe and highland conditions, where robustness, feed scarcity tolerance, and reproductive sufficiency are central production goals [1,2,3]. Akkaraman is most widely distributed across Central Anatolia and is typically characterized by white fleece and a strong association with extensive grazing systems, whereas Morkaraman is concentrated in Eastern Anatolia and is commonly distinguished by darker pigmentation and adaptation to harsher highland environments; both are generally considered multi-purpose under traditional production (primarily meat, with secondary milk and coarse wool) [4,5]. Population genetic analyses of Turkish sheep have consistently placed Akkaraman and Morkaraman among the closest related populations, supporting their use as a relatively similar comparative pair. In contrast, Zom sheep are described as a geographically restricted local population associated with the Karacadağ region of southeastern Türkiye, with reported morphometric descriptors and baseline reproductive/growth performance under regional husbandry, making the group comparatively more localized and potentially more differentiated for within-species cranial comparisons [6,7].

The sella turcica is a saddle-shaped depression on the dorsal surface of the sphenoid body within the middle cranial fossa, forming a central element of the cranial base [8,9,10]. Its rostral boundary is defined by the tuberculum sellae, its caudal boundary by the dorsum sellae, and its deepest portion, the fossa hypophysialis, houses the pituitary gland, thereby linking sellar morphology directly to the neuroendocrine axis [8,9,10]. Because the sellar region integrates with adjacent cranial base landmarks and lies in close topographic relationship to critical neurovascular anatomy, variation in sellar form has been treated as clinically and functionally meaningful in craniofacial and pituitary contexts; in humans, it is also widely used as a stable cranial base reference landmark in morphometric/cephalometric frameworks [11,12,13]. Recent veterinary CT-based work further shows that the sellar region can be modeled in 3D in ruminants and that measurable morphological differences exist at least across species, supporting its suitability for quantitative comparative analyses in domestic ungulates [14,15].

Three-dimensional geometric morphometrics quantifies complex anatomical form using configurations of homologous 3D landmarks, preserving geometric relationships that are often compressed or lost in linear or purely 2D descriptions [16,17]. In landmark-based analyses, the primary raw observations are the x-, y-, and z-coordinates of homologous anatomical points rather than single linear, angular, or perimeter measurements. Following Generalized Procrustes Analysis, translation, rotation, and scale are removed so that Procrustes coordinates represent shape variation alone, while centroid size provides a complementary global size metric derived from the same landmark configuration [16,17]. Breed and sex effects can then be evaluated using Procrustes ANOVA framed as high-dimensional linear models, and residual randomization permutation procedures provide distribution-free inference and pairwise testing that is well-suited to morphometric data structures [18,19]. Importantly, incorporating log-transformed centroid size allows explicit assessment of allometry (size and shape covariation), enabling breed differences in sellar shape to be interpreted both with and without size correction, an essential step when cranial base shape is expected to covary with overall cranial scaling [14,20,21].

Recent anatomical and morphometric evidence supports the sella turcica as a structurally informative region whose morphology shows meaningful variation and can be evaluated with quantitative shape approaches in clinical and craniofacial contexts [22,23]. In veterinary anatomy, emerging 3D modelling studies indicate that sella turcica morphology varies across ruminant species, while also emphasizing that within-species morphological classification and group-focused comparisons remain limited [14]. Building on these foundations and on recent 3D geometric morphometric applications to the pituitary fossa region [24], the present study quantified the sella turcica as a three-dimensional landmark configuration on CT-derived models. In this framework, overall size was summarized by centroid size, whereas shape was represented by Procrustes-aligned landmark coordinates. Accordingly, the aim of this study was to test whether sella turcica size and shape differ among three sheep breeds (including two relatively similar breeds and one more distinct breed) and between sexes, both in the raw shape space and after allometric (size) correction.

## 2. Materials and Methods

### 2.1. Study Sample and Osteological Collection

The cranial specimens used in this study were obtained from the osteological collection of the Osteoarchaeology Application and Research Center. According to the collection records, all individuals included in the study were older than 1 year, and sex information was available for every specimen. A total of 102 individuals were examined, representing three breeds: Akkaraman (*n* = 35; 18 females, 17 males), Morkaraman (*n* = 32; 14 females, 18 males), and Zom (*n* = 35; 16 females, 19 males).

### 2.2. CT Imaging and 3D Modelling

To access and reconstruct the sella turcica region without damaging the frontal part of the reference skulls, all specimens underwent computed tomography (CT) scanning specifically for this study (*n* = 102). CT examinations were performed at the Animal Hospital, Faculty of Veterinary Medicine, Istanbul University–Cerrahpaşa, using Siemens Somatom Scope vc30b and Siemens Somatom Sensation 16 systems (Siemens, Munich, Germany). A standardized protocol was applied across all specimens, with a slice thickness of 0.6 mm, 110 kV, 28 mA, and an acquisition time of approximately 14 s per specimen. Following image acquisition, segmentation and three-dimensional reconstruction of the sella turcica region were carried out in 3D Slicer (version 5.2.2), an open-source platform for medical image analysis [25]. The segmented target region corresponded to the sellar depression of the sphenoid body and included the tuberculum sellae, dorsum sellae, bilateral sellar margins, and the contour of the hypophyseal fossa, which together formed the 3D surface used for landmark digitization.

### 2.3. Landmark Definition and Digitization

**Figure 1:**
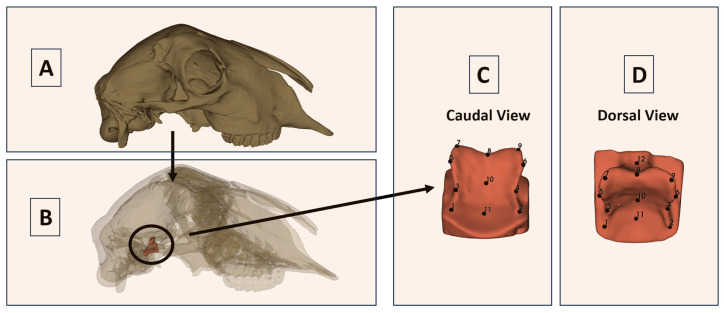
Localization, segmentation, and landmark configuration of the sella turcica. (**A**) Three-dimensional skull reconstruction showing the anatomical location of the sella turcica; (**B**) Transparent skull reconstruction indicating the segmented sellar region used for morphometric analysis (circled); (**C**) Isolated sella turcica in caudal view with numbered landmarks. In this view, LM1, LM3, LM5, and LM7 correspond to the left lateral side; LM2, LM4, LM6, and LM9 correspond to the right lateral side; LM8, LM10, and LM11 are median landmarks. LM12 is not visible in this orientation; (**D**) Isolated sella turcica in dorsal view with numbered landmarks. In this view, LM1, LM3, LM5, and LM7 correspond to the left side; LM2, LM4, LM6, and LM9 correspond to the right side; LM8, LM10, LM11, and LM12 are median landmarks. Landmark numbering corresponds to Appendix A, Table A1. Left and right refer to the anatomical sides of the specimen.

Following the 3D reconstruction of the sella turcica region, landmark data were collected by manual digitization in 3D Slicer (v5.2.2). Landmarking was performed directly on the isolated CT-derived 3D sellar surface. A total of 12 homologous anatomical landmarks were placed on each specimen to capture the geometry of the sella turcica, including its rostral boundary (tuberculum sellae), caudal boundary (dorsum sellae), bilateral lateral margins, and the midline/floor contour of the hypophyseal fossa (Figure 1). Landmarks were restricted to clearly identifiable anatomical points and digitized using standardized caudal and dorsal orientations to ensure homologous positions were consistently identified across all individuals. Thus, the recorded observations in the present study were the three-dimensional Cartesian coordinates (x, y, and z) of these 12 landmarks rather than isolated linear, angular, or perimeter measurements. The same landmark configuration was applied to all specimens, yielding a comparable coordinate dataset for subsequent geometric morphometric analyses. Operational definitions of all numbered landmarks are provided in Appendix A (Table A1).

### 2.4. Statistical Analyses

All statistical analyses were conducted in R [26]. Specimen labels were parsed to assign individuals to breed (Akkaraman, Morkaraman, Zom) and sex (Female, Male).

Centroid size (CS) was used as the global size metric of the 12-landmark sella turcica configuration. CS was defined as the square root of the sum of the squared Euclidean distances of all landmarks from their common centroid; therefore, it does not represent a perimeter or a single linear distance.

Differences in CS among breeds and sexes were tested using a two-way analysis of variance (ANOVA) with the model CS ~ breed × sex. When the main effect of breed was significant, Tukey’s HSD was used for post hoc pairwise comparisons. Model assumptions were evaluated by inspecting residual diagnostics, including Shapiro–Wilk tests and Q–Q plots for normality and Bartlett’s test for homogeneity of variances. Effect sizes were quantified using η^2^ based on sums of squares.

For shape analyses, 3D landmark configurations were aligned using Generalized Procrustes Analysis (GPA) to remove translation, rotation, and scale effects, yielding Procrustes shape coordinates. Following GPA, shape coordinates, Procrustes distances, and PCA scores are dimensionless (unitless), whereas centroid size retains the original metric unit of the landmark coordinates. Shape differences among breeds and sexes were assessed using Procrustes ANOVA implemented in the geomorph framework via residual randomization permutation procedures (RRPP; 1000 permutations). The primary model tested was shape ~ breed × sex, and significance was evaluated using permutation-based *p*-values. Pairwise differences in mean shapes among breeds were quantified using Procrustes distances and tested using permutation-based pairwise comparisons. To evaluate size-related shape change, allometry was assessed using the model shape ~ log (CS). In addition, a size-corrected model (shape ~ log (CS) + breed × sex) was fitted to determine whether breed and sex effects on shape persisted after accounting for allometric effects. The proportion of explained variance was reported as R^2^ from the RRPP models.

Morphological variation in shape space was summarized using principal component analysis (PCA) of Procrustes shape coordinates, and the percentage of variance explained by the first three components (PC1–PC3) was reported. PC scores were used descriptively to visualize group dispersion in morphospace (PC1–PC2 and PC1–PC3), and group-level trends were evaluated by comparing mean PC scores among breeds.

For visualization, the negative and positive extremes of PC1, PC2, and PC3 were reconstructed as estimated surface configurations from the PCA of Procrustes-aligned coordinates and displayed in dorsal and caudal views. These renderings were positioned schematically around the scatterplot to illustrate the direction of shape change along each principal component and do not represent additional specimens or exact specimen coordinates in morphospace.

## 3. Results

Results are presented separately for centroid size and overall sella turcica shape. Size comparisons refer to centroid size (CS), whereas shape comparisons refer to the Procrustes-aligned 12-landmark configuration of the sella turcica.

### 3.1. Size Differences Across Breeds and Sexes

Centroid size was evaluated using a two-way ANOVA with breed, sex, and their interaction (Table 1). Breed had a significant effect on CS (F (2,96) = 27.50, *p* < 0.0001, η^2^ = 0.356), whereas sex (F (1,96) = 0.673, *p* = 0.4142) and the breed × sex interaction (F (2,96) = 1.493, *p* = 0.2298) were not significant. Overall, Zom exhibited the largest centroid size (mean ± SD: 24.999 ± 2.928; median: 25.791), whereas Akkaraman showed the smallest centroid size (21.007 ± 2.269; median: 21.106). Morkaraman had intermediate values (21.365 ± 2.166; median: 21.465). Post hoc Tukey tests indicated that Zom was significantly larger than both Akkaraman and Morkaraman (*p* < 0.0001 for both comparisons), while Akkaraman and Morkaraman did not differ (*p* = 0.8257).

**Figure 2:**
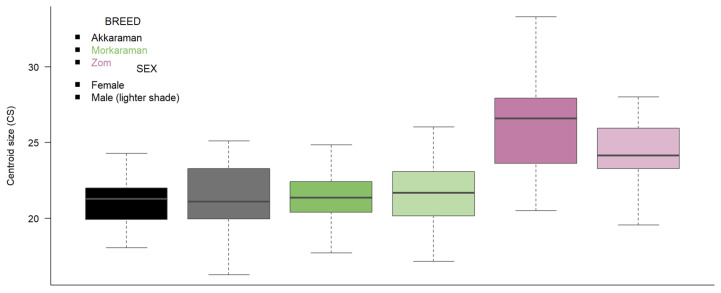
Breed differences in centroid size; females and males are shown separately.

Sex-specific comparisons within each breed showed no statistically significant differences between females and males after multiple-testing correction (FDR-adjusted *p*-values > 0.30 for all breeds). Nevertheless, the direction of the mean differences varied by breed: males were slightly larger in Akkaraman (Female: 20.833 vs. Male: 21.191) and Morkaraman (Female: 21.346 vs. Male: 21.379), whereas females were larger in Zom (Female: 25.850 vs. Male: 24.282), although this difference was not significant (FDR-adjusted *p* = 0.391 for *t*-test; 0.305 for Wilcoxon). These patterns are consistent with the boxplots, which show that the primary separation in CS is driven by breed, particularly the larger values observed in Zom, rather than by sex (Figure 2).

### 3.2. Shape Differences and Allometric Effects

Shape differences were evaluated from the Procrustes-aligned 12-landmark configurations of the sella turcica and tested with the Residual Randomization Permutation Procedure (RRPP; 1000 permutations). Thus, the comparisons in this section concern the overall three-dimensional shape of the sella turcica, as captured by the relative positions of all landmarks, rather than any single linear, angular, or perimeter measurement. In the first model, the effects of breed, sex, and the breed × sex interaction on overall sellar shape were assessed (Table 2). The analysis revealed a significant effect of breed on shape (R^2^ = 0.05068, F = 2.6682, *p* = 0.001). In contrast, the effect of sex was not significant (R^2^ = 0.01024, F = 1.0781, *p* = 0.361), and the breed × sex interaction was also not significant (R^2^ = 0.02731, F = 1.4376, *p* = 0.108). Overall, these results indicate that the average three-dimensional landmark configuration of the sella turcica differs among breeds, whereas sex does not produce a detectable shape separation in this sample.

To identify which comparisons drove the breed effect, pairwise tests were performed on the breed-mean Procrustes shapes of the sella turcica (Table 3). Here, Procrustes distance quantifies the magnitude of difference between the average Procrustes-aligned landmark configurations of two breeds; therefore, larger d values indicate greater overall three-dimensional shape dissimilarity of the sella turcica. These comparisons showed that Akkaraman and Morkaraman did not differ significantly (d = 0.04167, *p* = 0.360). In contrast, Zom differed significantly from Akkaraman (d = 0.08323, *p* = 0.001), representing the largest Procrustes distance observed, and Zom also differed significantly from Morkaraman (d = 0.05703, *p* = 0.032). Thus, breed-related shape variation was driven mainly by differences in the overall mean shape configuration of Zom relative to the other two breeds.

Allometric effects were examined using a separate model (Shape ~ log (CS)), which showed that log-transformed centroid size (CS) explained a significant portion of shape variation (R^2^ = 0.04077, F = 4.2508, *p* = 0.002). This finding indicates that part of the observed shape variation is associated with size-related (allometric) change.

To evaluate breed and sex effects independent of size, a size-corrected model (Shape ~ log (CS) + breed × sex) was fitted. In this model, the effect of log (CS) remained significant (R^2^ = 0.04077, F = 4.3760, *p* = 0.002). Importantly, the breed effect also remained significant after controlling for size (R^2^ = 0.03637, F = 1.9517, *p* = 0.009). In contrast, sex remained non-significant (R^2^ = 0.00989, F = 1.0609, *p* = 0.354). The breed × sex interaction showed only a marginal trend after size correction but did not reach statistical significance (R^2^ = 0.02779, F = 1.4914, *p* = 0.089). Taken together, these results suggest that shape variation in this dataset includes both an allometric component and a breed-specific component that persists beyond size effects, while sex-related shape differences are not evident.

Procrustes distance (d) represents the difference between breed mean Procrustes-aligned landmark configurations; higher values indicate greater overall 3D shape divergence.

### 3.3. Distribution of Specimens in Shape Morphospace

The PCA indicated that shape variation shows substantial overlap among groups rather than clear, discrete clustering. The first three principal components accounted for 48.02% of total shape variation (PC1 = 19.80%, PC2 = 17.22%, PC3 = 11.00%). Inspection of the score distributions revealed pronounced overlap among breeds across the PCA space.

**Figure 3:**
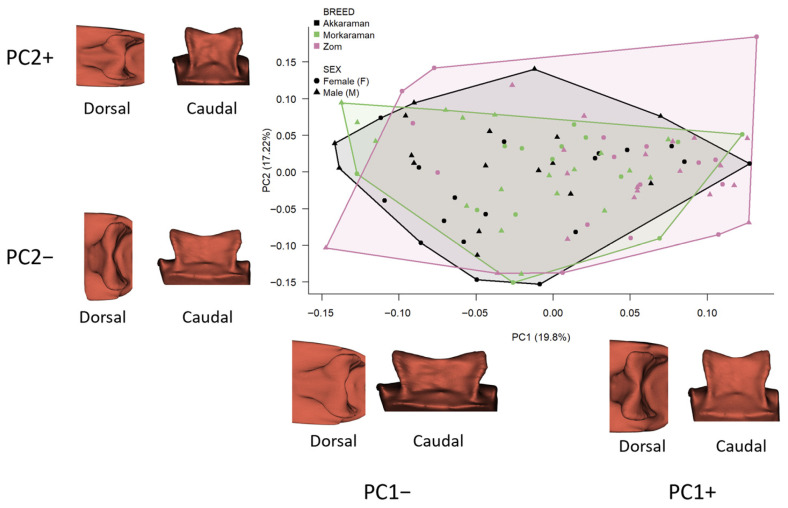
PC1–PC2 morphospace of sella turcica shape and schematic visualization of axis-associated shape extremes. The central scatterplot shows individual specimens plotted by their PC1 and PC2 scores from a PCA of Procrustes-aligned landmark coordinates. Colours indicate breed, and symbols indicate sex. The convex hulls summarize the distribution of the breed groups in morphospace. The surface renderings placed around the plot do not represent additional specimens or exact specimen coordinates. Instead, they illustrate the estimated shape configurations at the negative and positive extremes of PC1 and PC2 (PC1−, PC1+, PC2−, PC2+), each shown in dorsal and caudal views. Their placement around the plot is schematic and serves only to indicate the direction of shape change associated with each principal component.

In Figure 3, the surface renderings surrounding the PC1–PC2 scatterplot represent the estimated negative and positive shape extremes of PC1 and PC2 (PC1−, PC1+, PC2−, PC2+), each shown in dorsal and caudal views. Their placement around the graph is schematic and is intended only to indicate the direction of shape change along the axes, not the exact plotted position of any individual specimen.

Along PC1, the Zom group tended to have more positive scores on average (mean = 0.039; median = 0.055), whereas Akkaraman tended to be shifted toward more negative values (mean = −0.029; median = −0.044), with Morkaraman showing a more central distribution (mean = −0.011). However, because score ranges were broad within each breed, visual separation was not evident, and the distributions overlapped considerably. Nonetheless, a univariate test on PC1 scores indicated a statistically significant breed effect (ANOVA: F = 9.464, *p* = 0.000173), suggesting that differences along PC1 are better interpreted as a small but consistent shift rather than strong group discrimination (Figure 3).

**Figure 4:**
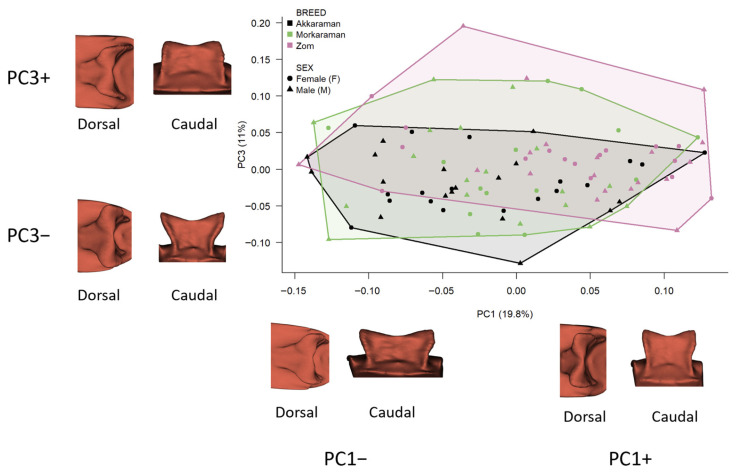
PC1–PC3 morphospace of sella turcica shape and schematic visualization of axis-associated shape extremes. The central scatterplot shows individual specimens plotted by their PC1 and PC3 scores from a PCA of Procrustes-aligned landmark coordinates. Colours indicate breed, and symbols indicate sex. The convex hulls summarize the distribution of the breed groups in morphospace. The surface renderings placed around the plot do not represent additional specimens or exact specimen coordinates. Instead, they illustrate the estimated shape configurations at the negative and positive extremes of PC1 and PC3 (PC1−, PC1+, PC3−, PC3+), each shown in dorsal and caudal views. Their placement around the plot is schematic and serves only to indicate the direction of shape change associated with each principal component.

For PC2, breed means were close to zero, and no systematic separation was observed (ANOVA: F = 0.125, *p* = 0.883), indicating that variation along this axis primarily reflects within-breed variation. Along PC3, a modest tendency was observed, with Zom showing more positive scores and Akkaraman more negative scores (Zom mean = 0.0177; Akkaraman mean = −0.0184), although overlap remained substantial. Even so, the breed effect on PC3 scores was statistically significant (ANOVA: F = 4.078, *p* = 0.0199). Overall, the PCA space does not support a sharp separation among breeds; instead, it suggests weak-to-moderate directional trends, particularly along PC1 and, to a lesser extent, PC3, superimposed on extensive overlap (Figure 4).

Across the principal component axes, inspection of the extreme shapes (dorsal and caudal views) indicates a clear reorganization of sella turcica morphology (Figure 3). Along PC1, the main change reflects overall proportions and the configuration of the hypophyseal fossa (pituitary fossa) opening. At positive PC1 values, the sella turcica becomes slenderer and more elongated (anteroposteriorly), whereas at negative PC1 values it appears mediolaterally broader and more compact. Concomitantly, the hypophyseal fossa opening changes in shape: at the negative PC1 extreme, the opening is wider anteriorly, while at the positive PC1 extreme this region becomes markedly constricted.

Along PC2, variation is expressed primarily in the dorsal view through the positioning of the lateral margins and in the relative breadth of the fossa entrance (Figure 3). At positive PC2 scores, the lateral borders are more medially constricted, producing a narrower “frame” of the sella outline, whereas at negative PC2 scores these lateral margins are more open laterally. In parallel, the hypophyseal fossa opening appears broader along this axis, suggesting that PC2 captures a “constriction–opening” pattern at the entrance region.

In addition, PC3 extremes indicate a more angular shift in the external contour geometry of the sella turcica (Figure 4). At positive PC3 values, the overall outline approaches a more square-like configuration, whereas at negative PC3 values the contour tends toward a shorter, low-height rectangular appearance. Taken together, these patterns suggest that PC1 primarily represents an axis of “elongation/slenderness versus mediolateral widening,” PC2 reflects “medial constriction versus lateral opening” of the margins, and PC3 captures a transition from a more square-shaped to a flatter rectangular contour.

Taken together, these PCA trends indicate that the compared breed differences concern overall sellar proportions and contour, particularly anteroposterior elongation versus mediolateral breadth, as well as the configuration of the hypophyseal fossa entrance, rather than a single localized feature.

## 4. Discussion

This study aimed to evaluate whether 3D sella turcica morphology provides a measurable basis for distinguishing among sheep breeds (including two relatively similar breeds and one more distinct breed) and whether sex-related differences are detectable when shape is quantified using geometric morphometrics. Overall, our results support the first part of this objective: breed was a significant determinant of sella turcica shape, whereas sex was not, and the breed × sex interaction was not statistically significant. Pairwise comparisons indicated that the observed breed effect was driven primarily by the Zom group, which differed significantly from both Akkaraman and Morkaraman, while Akkaraman and Morkaraman did not differ. Together, these findings suggest that sella turcica morphology can capture within-species, group-level differentiation, but that this differentiation is not necessarily expressed as a sharp morphospace separation. Together, these findings suggest that sella turcica morphology can capture within-species, group-level differentiation, but that this differentiation is not necessarily expressed as a sharp morphospace separation.

More specifically, the statistically supported breed signal appears to reflect coordinated differences in overall sellar proportions and contour rather than a single localized feature. Because the Zom group tended to occupy more positive PC1 scores and, to a lesser extent, more positive PC3 scores, its mean shape trend was associated with a more slender and anteroposteriorly elongated sella turcica, together with a relatively narrower and more constricted anterior entrance of the hypophyseal fossa. By contrast, the more negative side of PC1, toward which Akkaraman tended, corresponded to a mediolaterally broader and more compact configuration with a wider anterior opening. PC3 contributed a secondary contour difference: more positive values were associated with a more square-like external outline, whereas more negative values were associated with a flatter, lower-height rectangular appearance. Morkaraman generally occupied an intermediate position, which is consistent with the non-significant pairwise difference between Akkaraman and Morkaraman. Thus, the breed effect detected by Procrustes ANOVA and pairwise mean-shape comparisons should be interpreted as the cumulative result of proportional and contour shifts across the landmark configuration, not as a single isolated shape change.

An important aspect of the present dataset is that allometry contributed significantly to shape variation: centroid size (CS) showed a significant association with shape, indicating that part of the observed morphological variability reflects size-related change. Crucially, however, breed differences remained significant after controlling for CS, demonstrating that the breed signal is not solely a by-product of size effects. This size-corrected result strengthens the interpretation that breed-related variation reflects genuine shape differences in the sella region. In contrast, the sex effect remained non-significant in both the uncorrected and size-corrected models, suggesting that any sexual dimorphism in this structure is weak relative to within-breed variation in the present sample, or that sex differences, if present, may require larger samples or additional covariates (e.g., age class, body condition, skull size proxies) to be resolved.

Although inferential tests supported breed-related differences in shape, the PCA visualizations showed substantial overlap among groups, consistent with the small-to-moderate effect sizes observed in the Procrustes ANOVA. This pattern indicates that sella turcica morphology is informative for detecting breed-level trends, yet it may not provide high classification accuracy on its own without complementary anatomical regions or multivariate classification frameworks. Future work could therefore expand the anatomical context (e.g., incorporating adjacent cranial base landmarks), explore cross-validation-based classification, and test robustness across age strata or management conditions.

The inter-breed pattern indicates that this anatomical variability can also be captured at the intraspecific level: while the Zom group showed a clear separation, Akkaraman and Morkaraman showed largely overlapping distributions. Previous work on Turkish sheep breeds has shown that the close genetic clustering of Akkaraman and Morkaraman may help explain why shape differences between these two breeds remain limited [1]. In addition, long-term adaptation to low-input production systems and broadly comparable husbandry pressures may promote similar morphological patterns in relatively conservative cranial regions, such as the cranial base [2,3]. In contrast, Zom sheep are associated with a more localized breeding area in the Karacadağ region, and their reported morphological and performance characteristics suggest that the combined influence of environmental conditions, management practices, and potential differences in ancestry/admixture could contribute to a more differentiated cranial base geometry [6].

When the shape variation observed in the sella turcica is considered together with the relative positioning of the tuberculum sellae and dorsum sellae, which define the boundaries of the hypophyseal fossa, and with the reorganization of the cranial base contour, it suggests that the space provided within the endocranial cavity for pituitary gland accommodation and the potential spatial capacity may differ among individuals or groups [10,27]. The fact that the sella turcica can be reconstructed three-dimensionally in ruminants and can exhibit morphometric differences supports the view that this region constitutes an anatomically informative area for comparative analyses [14]. From a clinical perspective, documenting the normal range of breed-related sellar variation may aid the interpretation of CT or MRI studies of the skull base and pituitary region in sheep by providing a morphological baseline against which abnormal sellar morphology can be assessed. In addition, because the sella turcica is a centrally located and relatively protected cranial base structure, its 3D morphology may serve as an auxiliary trait in osteological, archaeozoological, or identification-oriented forensic contexts when more exposed cranial regions are incomplete or damaged, although these potential applications still require dedicated validation.

Allometry, the influence of size on shape, is a well-recognized component of morphological variation, and in complex regions such as the cranial base, part of the observed shape differences may arise directly from size-related change [21,28,29]. For this reason, comparisons based solely on raw shape can leave uncertainty as to whether group differences reflect genuine shape divergence or are primarily a by-product of size variation. In the present study, the size-shape relationship was explicitly evaluated, and the main findings remained consistent after accounting for size effects (i.e., in size-corrected analyses). In other words, the breed-related shape differences were not simply attributable to larger versus smaller individuals; the persistence of the same overall pattern after controlling for size supports the presence of a size-independent component of shape variation in sella turcica morphology, consistent with current Procrustes-based approaches for separating allometric and group effects in geometric morphometrics [19]. This strengthens the interpretation of our results by reducing ambiguity and indicates that the observed breed-related differentiation, particularly the separation of Zom from the other two breeds, reflects a robust morphological signal rather than a purely allometric effect.

One of the main limitations of this study is that, although all specimens are recorded as older than 1 year, their exact ages (in months/years) are not available. Age can influence cranial dimensions and certain anatomical contours while skeletal growth and maturation are still ongoing, and therefore, it could theoretically contribute to a portion of the observed morphological variation. However, in sheep, physical maturity and skeletal maturity do not necessarily occur at the same time: physical maturity is commonly linked to permanent incisor eruption within the second year of life, although the timing may vary with genotype and liveweight, whereas skeletal maturation in some elements may extend into a later period [30,31]. For this reason, while restricting the sample to individuals older than 1 year may not completely eliminate age-related variability, we consider that potential age effects are likely constrained with respect to the primary aim of the study, namely comparisons between breeds and sexes. Nevertheless, future studies would benefit from analysing specimens within narrower age classes and, where possible, incorporating precise age information to better evaluate any age-related contribution to sella turcica morphology.

## 5. Conclusions

This study demonstrates that the sella turcica, despite being a relatively small region of the cranial base, contains a measurable and biologically informative morphological signal in sheep. Using CT-based 3D reconstruction and geometric morphometrics, we found that both centroid size and shape vary among breeds, with breed-related shape differences primarily reflecting the separation of Zom from Akkaraman and Morkaraman. In contrast, no pronounced sex-related differentiation was detected in the present sample. Importantly, breed differences in shape persisted after accounting for size effects, indicating that the observed pattern is not solely driven by allometry. Collectively, these findings support the sella turcica as a useful comparative anatomical structure that may contribute to within-species and potentially broader taxonomic discrimination, and they highlight the value of integrating non-destructive CT imaging with 3D morphometric approaches for detecting subtle cranial base variation.

## Figures and Tables

**Table 1:** Results of the two-way ANOVA for centroid size (CS) showing the effects of breed, sex, and their interaction.

Effect	SS	MS	F	*p*-Value	η^2^
Breed	337.735	168.868	27.500	0.0000	0.3556
Sex	4.130	4.130	0.673	0.4142	0.0043
Breed × Sex	18.340	9.170	1.493	0.2298	0.0193

SS: Sum of Squares; MS: Mean Square; F: F-statistic; η^2^: Effect size.

**Table 2:** Procrustes ANOVA of overall sella turcica shape based on the 12-landmark configuration.

Effect	SS	MS	R^2^	F	Z	*p*-Value
log(CS)	0.11101	0.11101	0.04077	4.2508	3.4911	0.002
Breed	0.13798	0.06899	0.05068	2.6682	3.4551	0.001
Sex	0.02788	0.02788	0.01024	1.0781	0.3919	0.361
Breed × Sex	0.07434	0.03717	0.02731	1.4376	1.3038	0.108

**Table 3:** Pairwise comparisons between breed mean Procrustes shapes of the sella turcica.

Comparison	Procrustes Distance (d)	UCL (95%)	Z	*p*-Value
Akkaraman vs. Morkaraman	0.04167	0.05410	0.366	0.360
Akkaraman vs. Zom	0.08323	0.05351	3.740	0.001
Morkaraman vs. Zom	0.05703	0.05339	1.929	0.032

## Data Availability

The original contributions presented in this study are included in the article. Further inquiries can be directed to the corresponding authors.

## Appendix A. Landmark Definitions

**Table A1:** Definitions of the three-dimensional landmarks used for the geometric morphometric analysis of the sella turcica.

Landmark No.	Landmark Definition	Primary View Used for Placement/Confirmation
1	Left ventrolateral boundary point of the sellar surface, defined as the lowest point on the left lateral outline in the standardized orientation.	Caudal + dorsal
2	Right ventrolateral boundary point of the sellar surface, defined as the lowest point on the right lateral outline in the standardized orientation.	Caudal + dorsal
3	Left lower lateral margin point of the sellar wall.	Caudal + dorsal
4	Right lower lateral margin point of the sellar wall.	Caudal + dorsal
5	Left upper lateral margin point of the sellar wall.	Caudal + dorsal
6	Right upper lateral margin point of the sellar wall.	Caudal + dorsal
7	Left dorsolateral point on the dorsal/caudal sellar rim.	Caudal + dorsal
8	Midline point on the dorsal/caudal sellar rim, positioned between landmarks 7 and 9.	Caudal + dorsal
9	Right dorsolateral point on the dorsal/caudal sellar rim.	Caudal + dorsal
10	Midline point on the central floor of the hypophyseal fossa.	Dorsal (with caudal confirmation)
11	Midline point on the rostral floor of the hypophyseal fossa.	Dorsal (with caudal confirmation)
12	Midline apex of the dorsum sellae, defined as the highest point of the caudal sellar wall in dorsal view.	Dorsal
